# Challenges Facing Global Health Networks: The NCD Alliance Experience: Comment on "Four Challenges that Global Health Networks Face"

**DOI:** 10.15171/ijhpm.2017.93

**Published:** 2017-08-07

**Authors:** Katie Dain

**Affiliations:** The NCD Alliance, Geneva, Switzerland.

**Keywords:** Noncommunicable Diseases (NCDs), Civil Society Networks, Coalition-Building, Advocacy, Governance, Human Sustainable Development

## Abstract

Successful prevention and control of the epidemic of noncommunicable diseases (NCDs) cannot be achieved by the health sector alone: a wide range of organisations from multiple sectors and across government must also be involved. This requires a new, inclusive approach to advocacy and to coordinating, convening and catalysing action across civil society, best achieved by a broad-based network. This comment maps the experience of the NCD Alliance (NCDA) on to Shiffman’s challenges for global health networks – framing (problem definition and positioning), coalition-building and governance – and highlights some further areas overlooked in his analysis.

## Introduction

Shiffman’s ‘Four Challenges that Global Health Networks Face’^1^ is welcome and timely, given the ongoing proliferation of a web of global health networks, the democratisation of global health with a stronger and more meaningful role for civil society, and the complexity of tackling health issues that are deeply embedded within societies worldwide.

Chronic, noncommunicable diseases (NCDs)^ [1],2^ perhaps more than any other health issue, cry out for a broad-based alliance. The United Nations (UN) recognises that the prevention and control of NCDs cannot be achieved without taking a ‘whole-of-society approach.’^3^ And, while the term ‘NCDs’ covers a wide range of conditions, many share common drivers and solutions: uniting around a common agenda strengthens the case for action on all.

This commentary maps the experience of the NCD Alliance (NCDA: http://www.ncdalliance.org) onto the Four Challenges, and highlights some overlooked areas in Shiffman’s analysis^ [2]^.

## Why the Noncommunicable Disease Alliance?

In the early 2000s, it became clear that the NCD epidemic was an urgent global health and development challenge.^4^ NCDs account for 40 million (70%) of deaths globally, of which 16 million occur among the under-70s. Once considered ‘diseases of affluence,’ and omitted from the Millennium Development Goals (MDGs), the NCD burden often falls on the poorest in society. Indeed 87% of all premature NCD deaths occur in low- and middle-income countries.^5^ NCDs not only cause suffering for individuals and families, but also impede economic growth and overburden health systems. The tragedy is that there are cost-effective solutions to both prevent and treat NCDs.

The NCDA was formed in 2009 as the first global NCD civil society network with a central aim to raise NCDs up the political agenda by advocating for a UN High-Level Meeting on NCDs. This was achieved remarkably rapidly, in 2011 – and was only the second such Meeting to be held on a health issue (the first was HIV/AIDS in 2001).

Since then, NCDA advocacy has contributed to calls for global political commitments,^6^ notably the adoption by all governments of a set of NCD targets for 2025,^7^ and the inclusion of NCDs in the UN Sustainable Development Goals (SDGs),^8^ which set development priorities for the next 15 years. Over 50 national and regional NCD alliances have also emerged, reflecting and emulating the NCDA’s pioneering and effective model as a united platform for advocacy.

NCDA has demonstrated the value of working across diseases and risk factors for a common cause. What was a fragmented community has become a unified network of 2000 civil-society organisations from across the diverse NCD community, spanning 170 countries.

Since its inception, NCDA has identified and responded to numerous challenges – many identified by Shiffman – that have allowed it to consolidate and strengthen its position in global health.

## NCDA and Shiffman’s Four Challenges

### Framing

The first two of Shiffman’s challenges are focused on framing: ‘problem definition’ within a network, and external ‘positioning’ in the global health community and more broadly.

Over the years, NCDA has built consensus around a problem definition of NCDs that united the network at pivotal moments during global political processes, such as the evolution of the SDGs. There have been four primary elements of the narrative. First, NCDA aligned with the World Health Organization’s (WHO’s) ‘4x4’ definition of NCDs (four major risk factors and four major NCDs),^2^ while also recognising and reflecting the importance of the many co-morbidities linked to these four diseases through officially partnering with organisations that are leaders in mental health, oral health, osteoporosis and psoriasis. This was an important framing for advocacy and policy, as it provided governments with a prioritised, concise agenda, focusing on the four diseases that are responsible for 80% of premature NCD mortality and which share common risk factors and preventative strategies. Second, in the lead up to the 2011 UN High-Level Meeting on NCDs, NCDA framed NCDs as a human development priority, both regarding prevention (particularly the social determinants of health, including poverty, gender inequality and education) and equitable access and right to treatment and care. Third, the economic arguments for action versus inaction are integral to the definition, because the costs of failure to tackle NCDs are so high.^9,10^ Finally, NCDA has strongly focused on cost-effective, available solutions balancing both NCD prevention and treatment/care.

As Shiffman points out, ‘different positioning appeals to different audiences,’ and NCDA is now actively seeking to meaningfully involve people living with NCDs through “Our Views, Our Voices.” This global consultation will inform an Advocacy Agenda of People Living with NCDs to articulate the issues of most importance to those affected, their main recommendations for policymakers, and to understand how they would like to be involved in the NCD response. NCDA has also taken the opportunity afforded by the inclusion of NCDs in the SDGs to align and build allegiances with other sustainable development priorities well beyond health. This raises awareness among new audiences and donors – for example, in 2016 the Women Deliver conference included NCDs in its programme for the first time – and NCDA can be more responsive and nimble in reacting to changes in wider global development policy because it can leverage the knowledge and insights of these new connections.

### Coalition-Building

Shiffman’s third challenge – reaching beyond the obvious ‘core proponents’ of an issue to build a wider coalition – is particularly pertinent for NCDs. Different countries and communities face very different NCD challenges, and creating a society that supports the prevention and management of NCDs requires the involvement of ‘unusual suspects,’ for example sectors as broad as urban planning, agriculture, trade, employment, education, marketing, law, media, and many more.

NCDA has taken a phased approach to coalition-building. Initially, the alliance was established and governed by three international non-governmental organisation (NGO) federations – the International Diabetes Federation, the Union for International Cancer Control and the World Heart Federation– joined in 2010 by the International Union Against Tuberculosis and Lung Disease. Together, they represent the four major NCDs as defined by WHO. Their track record in global advocacy, good standing with the UN and WHO, extensive networks of experts and the cumulative reach of the federation’s members in over 170 countries built the legitimacy of NCDA.

This initial governing group was soon expanded by three more international federations,^11^ and over the years an extensive civil society network has been established for knowledge exchange and coordination on cutting-edge NCD advocacy, policy and practice. This network comprises professional societies, patient groups, academia, NGOs, and regional and national NCD alliances to name a few. NCDA has cultivated this network and acts as a crucial knowledge hub for multilingual advocacy and policy information, including by convening regular webinars to update the network on campaigns and share ideas, and a website with up-to-date information on processes and good practice. This knowledge sharing service is provided free of charge available to all, and is actively promoted among the network.

NCDA’s strategy and coalition-building efforts have recently evolved in two new directions. First, while NCDA has successfully brought together a wide range of NCD organisations, achieving the SDG target on reducing premature death from NCDs requires forging new alliances across the SDG agenda. NCDA is concentrating on four areas where strong evidence supports integration with NCDs: nutrition; HIV/AIDs; reproductive, maternal, newborn, child and adolescent health (RMNCAH); and environment. NCDA works together with like-minded alliances and organisations across these priority areas, such as PMNCH and Scaling Up Nutrition (SUN), to promote integrated solutions that bring co-benefits across these areas, making best use of limited funding and resources.

Secondly, the lack of progress on NCDs nationally and regionally has been criticised widely as “insufficient and highly uneven,”^12^ so NCDA has responded by scaling up its efforts to foster and strengthen the capacity of national civil society organisations and alliances. In 2015, only 33% of countries had a national NCD action plan or strategy, and only 31% had national NCD targets and indicators,^13^ and resources for implementation are scarce.^14^ NCDA’s efforts focus on building strong national NCD movements, developing programmes that focus on coalition-building and advocacy. In 2015 NCDA convened its first Global NCD Alliance Forum in Sharjah, UAE; the second will be held in December 2017. This will be attended by 300 leaders in NCD prevention and control, reinforcing NCDA’s position as a major convenor in the NCD community, and providing its network with opportunities for capacity building, networking and partnership forming in person, and planning and mobilising toward the 2018 UN High-Level Meeting on NCDs.

### Governance

Shiffman recognises that there are several different governance models for global health alliances – and NCDA’s governance has evolved to keep pace with its growing, maturing network and the landscape it operates in. For the first eight years, NCDA was an informal alliance governed by a Memorandum of Understanding between four (and, more recently, seven) international NGO federations. The network was coordinated by a small secretariat, which organised global advocacy, disseminated information, and significantly expanded the network.

NCDA’s relatively informal beginning enabled it to become established and to demonstrate its impact. From 2017, however, it will formalise and broaden out its governance, becoming a standalone NGO, registered in Switzerland, with a president, board and consolidated membership base. The new structure will provide more opportunities for members to engage in its work, and will facilitate the expansion of the membership, becoming more inclusive of the diverse organisations that are so important in tackling NCDs, across risk factors, diseases, ageing, mental health and sustainable development. This will strengthen the case for action and consolidate NCDA’s position as a leading and active player in global health.^15^

## Further Challenges for Noncommunicable Disease Networks

NCDA is a classic example of a successful network, bringing coherence and strength to a formerly diffuse community. However, a few aspects of NCDA’s experience were underdeveloped within Shiffman’s analysis.

- Framing – internally and externally – is indeed necessary, particularly given the wide range of stakeholders who need to be involved in tackling NCDs. But to be sustainable, a network cannot simply make the theoretical case: it must demonstrate that it *adds value* for its members, and achieves what its members could not achieve individually. The challenge here is that a network, by definition, does not operate on the frontline: the practical work (helping people to lead healthy lives and to treat those with NCDs) is carried out by network members, so the impact of an alliance is diffuse and sometimes hard to measure. Networks – particularly those focused on advocacy – need to find new and effective ways to *track and evaluate their impact* if they are to be sustainable.
- Closely linked is the issue of *funding*: donors may not fully understand the crucial importance of catalysing, coordinating, advocating and information-sharing across a wide membership. While catalytic funding for some health alliances has been forthcoming – notably tobacco control (Bloomberg/Gates Foundation) and networks focused on MDG priorities – there has been no equivalent for other NCD-relevant areas such as physical activity or alcohol. This challenge of funding for alliances appears to be reflected at national and regional level too.^14^
- A coalition-building challenge particularly pertinent for NCD civil-society networks is *conflicts of interest* of members. Should the private sector be permitted to fund a global health network, to become a member, or to be an active partner? From its inception, NCDA has engaged in and actively established partnership and collaboration with a range of stakeholders from all sectors, recognising that wide expertise and resources can help enact the change it seeks to achieve. Some private sector entities have a key role to play to support the implementation of NCD policies and to support countries in achieving NCD targets, and all companies can contribute to NCD prevention and management through workplace health programmes. NCDA (with appropriate safeguards) works with corporations as well as foundations and NGOs that can bring value to multi-sector partnerships, but does not partner with unhealthy commodity industries (ie, tobacco, alcohol, food and beverages).
- Shiffman does not sufficiently emphasise the role of *personal leadership* in effective civil society coalitions. Many alliances are established because of the vision and dedication of an inspirational, trusted and well-connected individual, around whom the network can coalesce. Ann Keeling, then-CEO of the International Diabetes Federation, recognised the opportunity for and potential impact of a civil-society NCD network in the mid-2000s that led to the formation of NCDA. This pattern has been frequently replicated in national NCD alliances, and across NCDA’s sister alliances in global health.
- Finally, Shiffman’s analysis could further stress the need to be *nimble and adaptable* – governing structures that allow networks to change rapidly with external circumstances. NCDA has evolved significantly (for example, shifting focus in 2015 from primarily global advocacy efforts to capacity building at national level), which has only been possible through regular independent reviews of strategy and governance.

## Ethical issues

Not applicable.

## Competing interests

Author declares that she has no competing interests.

## Author’s contribution

KD is the single author of the paper.

## Endnotes


[1] The 2013 WHO Global Action Plan on NCDs focuses on ‘four types of disease – cardiovascular diseases, cancer, chronic respiratory diseases and diabetes, which make the largest contribution to morbidity and mortality due to noncommunicable diseases, and on four shared behavioural risk factors—tobacco use, unhealthy diet, physical inactivity and harmful use of alcohol.’

[2] It is not the first time that NCDA has used Shiffman’s work – his theoretical framework on generating political priority has informed NCDA’s strategy and approach over several years [Keeling A. Using Shiffman’s political priority model for future diabetes advocacy. *Diabetes Res Clin Pract*. 2012;95:299-300. doi:10.1016/j.diabres.2012.01.011].

